# Rapid Field Immunoassay for Detecting Antibody to Sin Nombre Virus in Deer Mice

**DOI:** 10.3201/eid1310.070383

**Published:** 2007-10

**Authors:** Tony Schountz, Charles H. Calisher, Tiffany R. Richens, Audrey A. Rich, Jeffrey B. Doty, Mark T. Hughes, Barry J. Beaty

**Affiliations:** * University of Northern Colorado, Greeley, Colorado, USA; † Colorado State University, Fort Collins, Colorado, USA

**Keywords:** Sin Nombre virus, deer mouse, hantavirus, rapid diagnostic field test, EIA, rodent, PAGEIA, dispatch

## Abstract

We developed a 1-hour field enzyme immunoassay (EIA) for detecting antibody to Sin Nombre virus in deer mice (*Peromyscus maniculatus*). The assay specificity and sensitivity were comparable to those of a standard EIA. This test will permit identification of rodents with antibody to this and perhaps other hantaviruses.

Hantaviruses (family *Bunyaviridae*, genus *Hantavirus*) are rodentborne or insectivoreborne viruses; some are recognized causes of human hemorrhagic fever with renal syndrome or hantavirus pulmonary (or cardiopulmonary) syndrome (HPS) (*1*). The normal transmission cycle is rodent to rodent, without arthropod intermediate hosts. Each hantavirus has a single principal reservoir host, which suggests a coevolutionary relationship (*2*). In North America, the principal cause of HPS is Sin Nombre virus (SNV) because of the geographically widespread nature of its rodent host, the deer mouse (*Peromyscus maniculatus*), the most common mammal in North America.

As with other rodent reservoirs that harbor unique hantaviruses, most, if not all, deer mice become persistently infected without discernible pathologic consequences (*3*,*4*), which makes distinguishing infected from uninfected deer mice by simple observation impossible. Development of a field-relevant technique for detection of antibody to SNV would be of value; the technique could be exploited for further investigations of the virus–reservoir host interactions and characteristics and to determine whether experimental infections of deer mice with SNV accurately parallel natural infections (*3*,*4*).

Commonly used serologic tests for deer mice require a minimum of 3–5 hours to complete (*2*,*5*,*6*) and thus are impractical to use in the field in a single day without putting the rodents at risk for death from heat, cold, dehydration, trap injuries, and other hazards while tests are being conducted. We modified a previously described protein-A/G horseradish peroxidase enzyme-linked immunosorbent assay (PAGEIA) to detect antibodies to SNV in deer mice (*7*). The test can be completed in ≈1 hour under relatively primitive field conditions. The assay has advantages over more laborious assays used for similar purposes and, because it is mammal-specific rather than species-specific, we expect this assay will be applicable to serologic tests of mammals of many other species.

## The Study

A fragment of the S segment (nt 43–394) encoding part of the nuclecapsid was cloned into pET21b with a C-terminal His tag to produce a 15-kDa truncated antigen (*8*) for use in the assay. Deer mice were trapped near Fort Lewis, Colorado, and blood was collected as previously described (*9*); whole blood was diluted (1:100) in 1 mL of phosphate-buffered saline (PBS) in 96 deep-well plates (P-DW-11-C, Axygen, Union City, CA, USA) at time of collection to expedite sample loading. The remainder of the blood was frozen on dry ice and returned to the laboratory for additional testing.

Wells of 96-well polyvinyl chloride plates (Falcon 353912, BD Biosciences, San Jose, CA, USA) were coated with 100 µL of 2 µg/mL recombinant nucleocapsid in PBS and blocked (0.25% gelatin in PBS) a week in advance. Wells were washed in the field 3× with 200 µL of PBS (pH 7.0) by using an 8-channel pipettor, and blood in PBS was added from the deep well plate; positive and negative (1:100) controls (diluted in PBS) were included. Plates then were incubated at ambient temperature (range ≈23°C–29°C) for 30 min. After 3 more washes with PBS/0.5% Tween-20, 100 µL of pretitrated staphylococcal protein-A/streptococcal protein-G horseradish peroxidase conjugate (Pierce Biotechnology, Inc., Rockford, IL, USA) diluted 1:1,000 in PBS was added for 30 min. Plates again were washed 3× with PBS-Tween-20, and 100 µL of activated ABTS substrate was added to each well. After 15 min of incubation at ambient temperature, wells were scored by using a 0–4+ system, with 0 indicating no reaction (i.e., clear, no color) and 4+ representing the strongest signal (i.e., dark green color). Samples deemed 1+, 2+, 3+, or 4+ were considered positive (very weak, weak, strong, very strong, respectively). Samples were retested under laboratory conditions with PAGEIA and standard Centers for Disease Control and Prevention (CDC) enzyme immunoassay (EIA) (*5*).

Blood samples from 222 deer mice were collected during 3 trapping sessions in the summer of 2006, and 39 samples were scored as positive in the field by PAGEIA; 183 were negative by the field PAGEIA, repeat laboratory PAGEIA, and the standard EIA in the laboratory. One sample (HA-2564) was scored negative by field and laboratory PAGEIA, but (low) positive (optical density [OD] of 0.327) by conventional EIA (Table).

**Table:** Comparison of results of PAGEIA and standard EIA for detection of antibody to Sin Nombre virus (SNV) in blood samples from 40 deer mice captured in southwest Colorado, 2006*

Accession no.	Field PAGEIA score†	Laboratory PAGEIA OD‡	Laboratory PAGEIA score§	Standard EIA OD¶	Standard EIA score#
HA-2548	3+	1.254	Pos	0.903/0.066	Pos
HA-2552	4+	2.406	Pos	1.083/0.113	Pos
HA-2554	4+	1.788	Pos	1.395/0.058	Pos
HA-2558	4+	2.383	Pos	1.462/0.055	Pos
HA-2560	4+	1.913	Pos	1.378/0.086	Pos
HA-2564	0	0.001	Neg	0.327/0.055	Pos
HA-2565	1+	0.236	Pos	0.715/0.046	Pos
HA-2567	4+	2.123	Pos	1.485/0.080	Pos
HB-2604	4+	2.065	Pos	1.161/0.067	Pos
HB-2608	2+	0.855	Pos	0.653/0.095	Pos
HB-2612	1+	0.282	Pos	1.136/0.071	Pos
HB-2616	1+	0.311	Pos	0.458/0.001	Pos
HB-2617	2+	0.517	Pos	0.819/0.008	Pos
HB-2618	2+	0.494	Pos	1.085/0.009	Pos
HB-2622	3+	1.254	Pos	1.519/0.029	Pos
HB-2628	1+	0.493	Pos	0.220/0.082	Neg
HB-2630	3+	1.609	Pos	0.681/0.008	Pos
HA-2570	4+	1.970	Pos	0.389/0.024	Pos
HA-2578	4+	2.101	Pos	1.185/0.017	Pos
HB-2642	4+	2.784	Pos	1.294/0.063	Pos
HA-2601	4+	2.699	Pos	0.921/0.121	Pos
HA-2609	2+	0.608	Pos	0.228/0.042	Neg
HA-2612	4+	2.482	Pos	1.072/0.085	Pos
HA-2616	1+	0.331	Pos	0.076/0.059	Neg
HB-2681	3+	0.977	Pos	1.392/0.048	Pos
HB-2682	3+	1.095	Pos	1.326/0.042	Pos
HA-2634	4+	3.010	Pos	0.863/0.014	Pos
HA-2647	4+	2.824	Pos	0.720/0.023	Pos
HA-2666	4+	2.682	Pos	0.477/0.028	Pos
HB-2706	1+	0.836	Pos	0.324/0.032	Pos
HB-2710	2+	0.664	Pos	0.155/0.035	Neg
HB-2712	3+	1.599	Pos	0.345/0.033	Pos
HB-2717	1+	1.098	Pos	0.293/0.027	Pos
HB-2720	3+	2.581	Pos	0.630/0.039	Pos
TS-0830–6	2+	0.889	Pos	0.799/0.097	Pos
TS0830–7	1+	0.000	Neg	0.030/0.033	Neg
TS-0830–8	3+	2.014	Pos	0.800/0.024	Pos
TS-0830–9	4+	1.949	Pos	0.884/0.054	Pos
TS-0830–18	4+	2.112	Pos	1.180/0.021	Pos
TS-0830–20	3+	1.427	Pos	0.820/0.072	Pos

*PAGEIA, protein-A/G horseradish peroxidase enzyme-linked immunosorbent assay; EIA, enzyme immunoassay; OD, optical density; Pos, positive; Neg, negative. †Field scores were based upon visual inspection without instrumentation, with 0 as negative, 1+ as very weak, 2+ as weak, 3+ as strong, and 4+ as very strong, relative to positive and negative control samples. ‡Field-collected samples were retested by PAGEIA under laboratory conditions and the OD reported here. The instrument was blanked on the negative control sample. §OD >0.200 above the negative control was considered positive. ¶For laboratory EIA, OD was recorded for diluted (1:100) blood with both SNV antigen (numerator) and control antigen (denominator). #A sample tested with SNV antigen and having an OD >0.300 was considered positive if the OD of that sample with the control antigen was <0.150. In regard to sample HBV-2717, the OD of the laboratory EIA with antigen was 0.293, very near the acceptable minimum, and the background was 0.027, which is very low; this sample was considered provisionally positive.

Of the 39 samples that were scored positive in the field, 5 discrepancies between these and laboratory tests were found (Table). One sample (TS-0830–7) scored as 1+ in the field was determined to be negative on subsequent laboratory testing by both PAGEIA and conventional EIA. The other 4 samples (HB-2628, HA-2609, HA-2616, HB-2710) were scored as positive by field and laboratory PAGEIA but negative by conventional EIA. In the field, each of these samples was scored as 1+ or 2+ and had ODs of 0.331–0.664 by laboratory PAGEIA. However, ODs ranged from 0.076 to 0.228 by conventional EIA.

The PAGEIA results were similar to results of conventional EIA, with a specificity of 82.9% (184 negatives/222 total rodents) versus 84.7% (188/222) for conventional EIA. The sensitivity of the PAGEIA was 97.1% (34 positive by PAGEIA/35 positive by conventional EIA).

## Conclusions

We have modified an existing serologic assay so that it is suitable for use in the field. The assay relies on a staphylococcal protein-A and streptococcal protein-G horseradish peroxidase conjugate (*10*). Each protein has the capacity to bind to the Fc portions of antibodies, including immunoglobulin M (IgM) and IgA for protein A (*11*,*12*), but has highest affinity for IgG subclasses of many mammalian species.

All samples scored 3+ or 4+ were also positive in laboratory tests when results were read by using a spectrophotometer. Thus, we are confident that such samples in the field will indicate seropositive animals. Because we are suggesting that this assay be used for identifying seropositive rodents and not for determining seroprevalence (although it appears to be adequate for those studies as well) and to be conservative, we considered only samples that appeared dark green (3+ and 4+) in the field assay to be positive with relative certainty. To minimize the complexity of the PAGEIA under field conditions, we did not use a negative control antigen to assess nonspecific reactivities of serum samples. Use of this test will allow deer mice with antibody to SNV to be identified. Deer mice are the population most likely to be naturally infected with that virus, and those rodents can be retained for further testing and for studies of tissues, live cells, and body fluids to be used for subsequent laboratory investigations, such as for determining cellular immunologic responses, viremia levels, viruria levels, and virus shedding in excreta and secreta.

Additional limitations of the PAGEIA are similar to those of other serologic tests. PAGEIA can detect only seropositivity, which is not necessarily indicative of current infection or of current shedding of virus. It also binds only with high affinity to IgG; thus, it is not useful for discriminating other immunoglobulin classes, such as IgM, the presence of which usually indicates recent infection.

Because of the broad mammalian species specificities of a protein-A and protein-G conjugate, the rapid PAGEIA likely can be used to test for antibodies to other antigens in other mammals. Lee et al. (*7*) characterized the reactivities of protein A and protein G with IgG from rodents of several species. They found that serum specimens from both sigmodontine rodent species (deer mice and hispid cotton rats, *Sigmodon hispidus*) they tested were recognized by protein-A and/or protein-G conjugates. Similar laboratory-based PAGEIAs have also been used to detect antibody to antigens of agents causing other infectious diseases, including severe acute respiratory syndrome coronavirus-like viruses and Nipah virus in bats (*13*–*15*).
